# Selectivity and Longevity of Peripheral-Nerve and Machine Interfaces: A Review

**DOI:** 10.3389/fnbot.2017.00059

**Published:** 2017-10-31

**Authors:** Usman Ghafoor, Sohee Kim, Keum-Shik Hong

**Affiliations:** ^1^School of Mechanical Engineering, Pusan National University, Busan, South Korea; ^2^Department of Robotics Engineering, Daegu Gyeongbuk Institute of Science and Technology, Daegu, South Korea; ^3^Department of Cogno-Mechatronics Engineering, Pusan National University, Busan, South Korea

**Keywords:** peripheral-nerve, longevity, neuroprosthetics, selectivity, amputation, nerve-machine interfaces, stability, electro-neurographic signals

## Abstract

For those individuals with upper-extremity amputation, a daily normal living activity is no longer possible or it requires additional effort and time. With the aim of restoring their sensory and motor functions, theoretical and technological investigations have been carried out in the field of neuroprosthetic systems. For transmission of sensory feedback, several interfacing modalities including indirect (non-invasive), direct-to-peripheral-nerve (invasive), and cortical stimulation have been applied. Peripheral nerve interfaces demonstrate an edge over the cortical interfaces due to the sensitivity in attaining cortical brain signals. The peripheral nerve interfaces are highly dependent on interface designs and are required to be biocompatible with the nerves to achieve prolonged stability and longevity. Another criterion is the selection of nerves that allows minimal invasiveness and damages as well as high selectivity for a large number of nerve fascicles. In this paper, we review the nerve-machine interface modalities noted above with more focus on peripheral nerve interfaces, which are responsible for provision of sensory feedback. The invasive interfaces for recording and stimulation of electro-neurographic signals include intra-fascicular, regenerative-type interfaces that provide multiple contact channels to a group of axons inside the nerve and the extra-neural-cuff-type interfaces that enable interaction with many axons around the periphery of the nerve. Section Current Prosthetic Technology summarizes the advancements made to date in the field of neuroprosthetics toward the achievement of a bidirectional nerve-machine interface with more focus on sensory feedback. In the Discussion section, the authors propose a hybrid interface technique for achieving better selectivity and long-term stability using the available nerve interfacing techniques.

## Introduction

**Figure 1:**
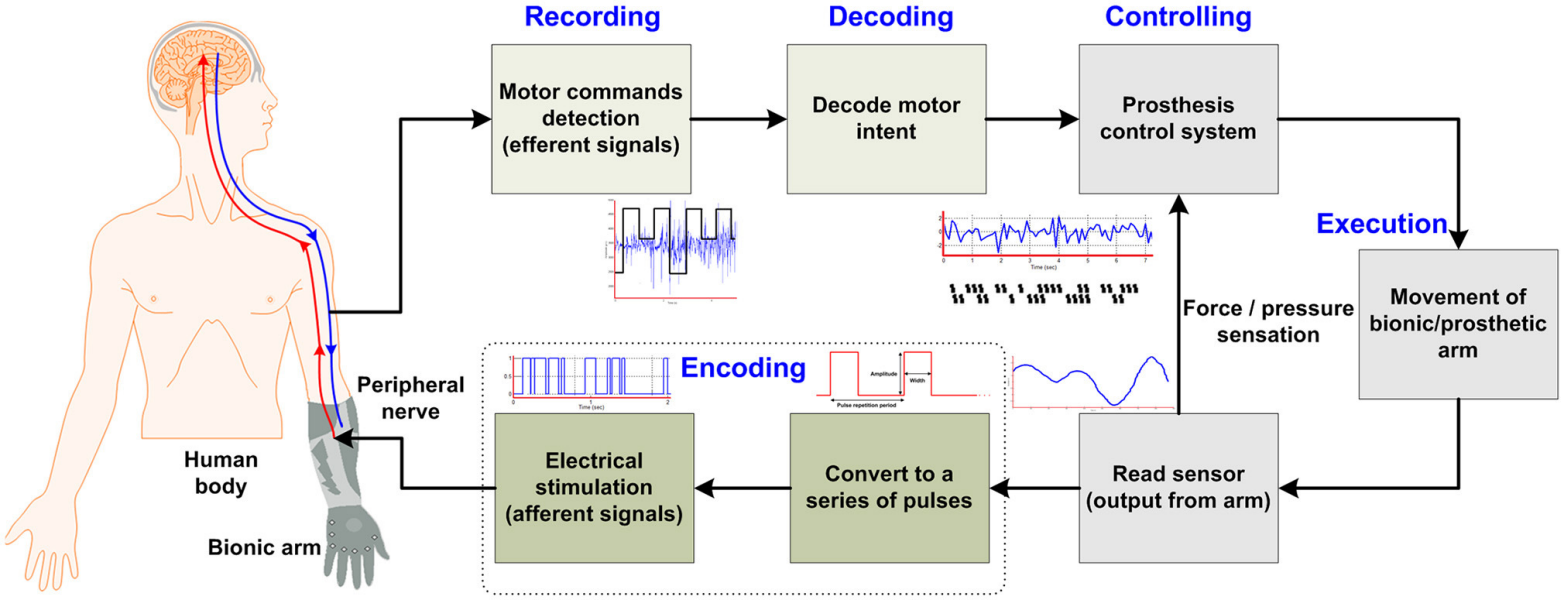
Concept of bi-directional control for bionic arm systems.

According to Ziegler-Graham et al. (2008), approximately 2 million people in the United States have suffered from the loss of limb(s). Substantial progress has been made in the form of highly sensorized cybernetic prostheses for restoration of the sensorimotor functionalities of limbs for those who have undergone an amputation. Achieving an effective interface between the nervous system and prostheses takes a long time due to the limitations of necessary components. The recent research in prosthetic technologies reveals a trend toward natural bidirectional communication between the amputee and the bionic arm/prosthesis and subsequently possible solutions in developing such a prosthesis that enables sensory restoration. The ultimate goal is to develop a bidirectional interface between the nervous system and a given prosthesis (e.g., a bionic hand, arm, or leg). This can be achieved using the form of a closed-loop control that sends motor commands on efferent pathways and returns the sensory feedback on afferent pathways by means of stimulation. According to several studies (Micera et al., 2008; Rossini et al., 2010; Raspopovic et al., 2014), an ideal closed-loop bidirectional prosthesis-user interface has the following mandatory components (see Figure 1 below): (i) peripheral-nerve data-recording electrodes; (ii) decoding of user intention; (iii) production of motor commands for the prosthesis system; (iv) passage of this information to the controller for controlling the speed/force for handling an object; (v) sensors embedded in the bionic hand/arm to capture the environmental information; and (vi) a sensory subsystem to encode the feedback to an amputee through a nerve stimulator (stimulation electrodes), which evokes the sensation produced through a contact with the manipulated object. Particularly for those who have lost upper limbs, natural sensory feedback through the prostheses is especially desired (Biddiss et al., 2007; Pylatiuk et al., 2007; Wijk and Carlsson, 2015). Witteveen et al. (2012, 2015) found that sensory-feedback systems improved the control of prosthetic hands, eradicated the unpleasant phantom limb pain sensation (Flor et al., 2001), and enhanced the sense of self-esteem. Object discrimination tasks were successfully accomplished by an amputee using artificial tactile and proprioceptive feedbacks without visual or auditory cues (Horch et al., 2011).

Lost functions were restored to the patients with central nervous system damages by electrically activating the intact tissues distal to neural lesions. This kind of activation can be realized via interfaces positioned either on the body surface or on the muscles near the motor points, or can be directly implanted in the motor nerves (Kuiken et al., 2004; Clemente et al., 2016; Davis et al., 2016; Patel et al., 2016). Nerve-fiber recording and stimulation from the nervous system have been carried out with the help of electrodes (Leventhal and Durand, 2003). Over the past 70 years, peripheral nerve interfaces enabling the restoration of sensorimotor functions have been improving and evolving (Branner and Normann, 2000). Now, with these systems, researchers are able to record the activity of the nerves elicited by volitional movements and electrically stimulate the peripheral nerves to move muscles. Direct nerve stimulation has several advantages over alternative methods, which include the low power consumption, multiple-muscle control via a single implantation, and the inherent capability of positioning the interface away from the contracting muscles (Koole et al., 1997). Peripheral-nerve electrodes, to be effective in neuroprosthetic applications, must have the capacity of targeting specific axons for activation without stimulating others. Moreover, they must be safe and biocompatible, and have stimulation characteristics that will remain stable over many years. This review mainly focuses on the amputees having limb loss(es) (not those having spinal cord injuries), because the scope of this review is restricted to peripheral nerve interfaces: In those amputees who lost only sensory end organs, the nerves connecting the end organs to the brain are functioning normally.

The rapid development of bidirectional peripheral-nerve interfaces has made available several methods for recording of motor commands from nerves and/or stimulation on nerve fibers. The main characteristics in bidirectional interfaces are the selectivity of axons and the degree of invasiveness. Achieving an optimal effectiveness entails a tradeoff between the reduced invasiveness for stability and the sufficient invasiveness for greater selectivity, in which the latter might damage the nerve and also affect chronic stability. For applications, an interface involving multiple electrodes implanted in an amputee's peripheral-nerve should be able to evoke a sensory response, record motor commands, and control multiple limb functions. Micera et al. (2008) suggested that the first step toward a suitable reproduction of sensorimotor functionality is to determine the topographical location in the nerve for desired functions. According to Gustafson et al. (2009) and Badia et al. (2010), the identification of fascicular contact points for electrode positioning is absolutely essential to selective interfacing with individual fascicles in a particular nerve. A various types of devices to improve the nerve-machine interface for the restoration of lost neural functions can be pursued. However, the long-term stability and selectivity of neural recording and stimulation remains a challenge. A key for an advanced neuro-prosthetics is the achievement of selective sensory perception, durability, and natural discernment from the nerve-machine interface. The selectivity, longevity, and long-term stability are absolutely an integral part for the establishment of long-lasting and functional sensory feedback for an amputee (Warren et al., 2016; Wurth et al., 2017).

**Figure 2:**
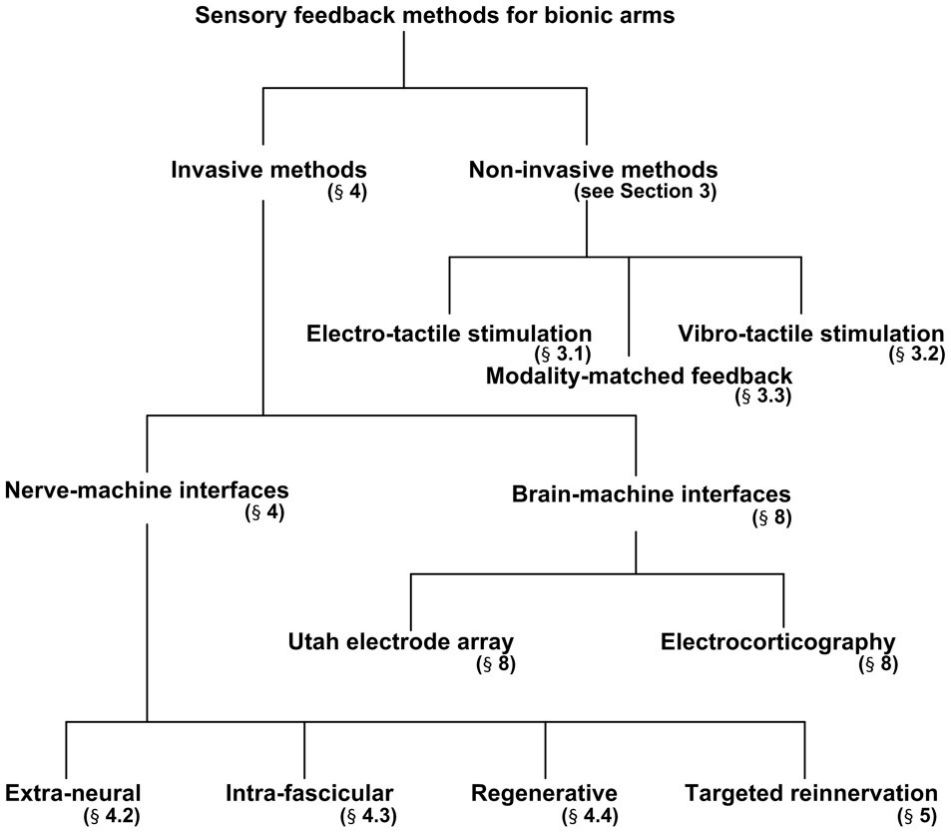
Breaks down of sensory feedback methods used in bionic arm systems.

The aim of the present review is to discuss the invasive and non-invasive methods in detail for evoking sensory feedback and recording intended motor commands from peripheral nerves. The authors first briefly outline the structure of the peripheral nerves that are responsible for transmitting motor commands and sensory feedback signals from/to the brain. Second, three different methodologies for evoking sensory feedback will be discussed: (i) Indirect (non-invasive) elicitation feedback by applying pressure or current, (ii) direct (invasive) elicitation through the peripheral nerve using several types of interfaces, and (iii) a cortical stimulation method to activate the somatosensory cortex in the brain. Figure 2 breaks down the methods discussed in this paper for providing sensory feedback to a bionic prosthesis. We will also discuss two hybrid techniques for bidirectional control of a bionic arm from the perspective of selectivity and longevity. Finally, in section Discussion, we propose a scheme for enhanced selectivity, longevity, and long-term stability for sensory feedback using the current techniques available in the field.

## Structure of peripheral nerves

The roots of the peripheral nervous system lie within the spinal cord, and the axons spread inside the peripheral nerves to reach the target organs. In this way, the peripheral nervous system fulfills its chief responsibility; the transmission of information between the brain and the extremities. In the peripheral nerves, numerous motor (called efferent) and sensory (called afferent) fibers are present (Johnson et al., 2000; Fisher et al., 2014; Mildren and Bent, 2016). The afferent fibers ranging 2–20 μm in diameter, either myelinated or unmyelinated, terminate at specialized sensory receptors in the skin, tissues and muscles. These fibers are responsible for sending mechanical, thermal, and noxious stimuli to the brain. Efferent motor fibers, on the other hand, send movement/motor commands from the brain to muscles. Both afferent and efferent nerve fibers are clustered individually in the form of fascicles that are surrounded by connective tissues in the peripheral nerves. In addition to the bundles of nerve fibers, three supportive sheaths bind the fibers in an organized structure (namely, epineurium surrounding the nerve, perineurium surrounding the fascicle, and endoneurium surrounding the fiber). Several researchers over the years have developed interfaces for accessing multiple and distinct levels of the nerves by penetrating the protective sheaths longitudinally and/or transversally. In this review, we will focus more on afferent fiber interface techniques for provision of sensory feedback.

### Afferent receptors

Touch, pressure, proprioception, temperature, and pain fall under the rubric of somatic sensation. The somatic receptors are divided according to the following broad categories: (i) proprioceptors that provide information on joint position and motion; (ii) nociceptors for temperature, pressure, different chemical stimuli, and a combination of these; (iii) thermoreceptors for mild temperature; (iv) cutaneous mechanoreceptors for touch and pressure, and (v) chemoreceptors for detection of certain chemical stimuli. Several types of sensory receptors are available in the body, and their quantity varies with respect to their body location. According to Johansson and Vallbo (1979), the most innervated parts in the body are hands, which permit fine manipulation and precise perception of the environment. As an illustration of a human finger's utility, the fingertip has approximately 241 units/cm^2^ of cutaneous mechanoreceptors for very fine sensory resolution, as compared with the palm that has 58 units/cm^2^ only. The important sensory feedbacks required for prostheses are proprioception and tactile sensation. For instance, cutaneous mechanoreceptors are essential for executing daily routines and simple tasks such as the grasping a glass. Johnson et al. (2000) and Johansson and Flanagan (2009) have demonstrated that detection of minute slippage of an object from a hand is by Meissner corpuscles and rapidly adapting afferents; to avoid slippage, a reflexive force is triggered. This type of testing, therefore, might be useful for assessment of the complete functionality of a rapidly adapting system. In the past, several approaches have been used to test and provide sensory feedback to bionic arms/prostheses. In the next section, non-invasive methods utilized for indirect sensory feedback are discussed.

## Non-invasive methods of sensory feedback

Several substitutions for sensory perception have been developed, which do not require implantable interfaces (Lundborg and Rosen, 2001; Visell, 2009; Khasnobish et al., 2016): Sensory substitution is a technique to provide an alternative path for necessary sensory information to the body using other sensory passages that are different from those naturally used. For instance, Kaczmarek et al. (1991) revealed that hearing and vibration can serve as a substitute for touch and pressure, respectively. Due to the essential need to restore physiological sensory information for those who have gone through a traumatic event and/or amputation, many of these sensory substitutions have been employed for battery-powered prostheses to achieve sensation. However, these modalities are not yet robust or effective enough to be applicable to routine tasks of daily living. Their use in commercially available prosthetics has not yet been widely adopted; however, they have been successful in controlled environments with certain limitations. The commonly used techniques are electro-tactile and vibro-tactile sensory substitutions as well as modality-matched feedback, which use an electric current and mechanical vibration in the residual skin area of the limb. This can help in encoding information on object manipulation, grasping force, elbow angle, and approaching direction (Hsiao et al., 2011; Chen et al., 2016; Clemente et al., 2016; Isakovic et al., 2016; Xu et al., 2016).

### Electro-tactile stimulation

The electro-tactile sensory modality is a method of passing electrical current through the skin of an amputee to elicit perception (Yem and Kajimoto, 2017). The sensation is not necessarily confined to the zone under the stimulating device, and the elicited sensations can spread if these are placed near the nerve bundles. Electro-tactile (electrocutaneous) stimulation is either voltage- or current-regulated. The use of voltage-regulated stimulation can minimize the chance of skin burns. Visell (2009) showed that the change in impedance and load at the site of interface might not affect the value of current in electro-tactile-current-regulated stimulation. Multiple features of this modality can be controlled to elicit sensory percepts. These features include (i) the place, material, and geometrical properties of the interface, (ii) the parameters of the current (duration, frequency, and amplitude), and (iii) end-organ thickness and location of the skin stimulated (Boldt et al., 2014; Hartmann et al., 2015; Paredes et al., 2015; Strbac et al., 2016). Subjects often describe electro-tactile sensations, qualitatively, as burning, pain, touch, pinch, tingle, pressure, vibrations, itch, sharp, etc. depending on the stimulation voltage or current. Although electro-tactile feedback cannot replicate direct forces and special types of touch sensations yet, it can produce a wide range of tactile sensations. Also, electro-tactile stimulation has been used for providing an alternative way to visual perception to the blind (Kaczmarek et al., 1991). The experimental study with electro-tactile feedback by Perovic et al. (2013) showed its suitability for haptic perception as well. Their results revealed that electro-tactile feedback has a potential for delivering haptic sensations from devices such as prosthetic hands. The users of this modality have been able to regulate the grasping forces as well as to the angular displacement of the prosthetic hand to predefined levels (Clemente et al., 2016; Schweisfurth et al., 2016). Further, Patel et al. (2016) improved the control performance by the grasping function by providing tactile stimulations to individual fingers (multiple degrees of freedom) in comparison to open-loop controls (Jorgovanovic et al., 2014; Dosen et al., 2015; Isakovic et al., 2016; Xu et al., 2016). Brain-computer interface (BCI) is a method of communication between brain and hardware by means of signals generated from the brain without the involvement of muscles and peripheral nerves (Naseer and Hong, 2013, 2015; Naseer et al., 2016; Rutkowski, 2016). An opposite modality of it is known as BrainPort, which has been designed to support a direct link from a computerized environment to the human brain non-invasively (Danilov and Tyler, 2005). In the application of electro-tactile stimulation (Tyler et al., 2003), the BCI and a head-mounted accelerometer could be served as a vestibular substitution that uses the unique patterns of electrotactile stimulation on the tongue for restoration of head-body postural coordination. Most types of electro-tactile stimulation are percutaneous (direct stimulation of the nerves) and transcutaneous (through the skin) stimulations. A major disadvantage of the percutaneous type of electro-tactile stimulation is the additional distress or pain to the patient. High voltage stimulation is required in stimulation through the skin without any insertion leads because of high impedance of the dry skin. This can be considered as a disadvantage of the transcutaneous electro-tactile stimulation. However the advantage of these types of feedback is the requirement of relatively simple circuits and electrodes placed on the skin, which can considerably reduce both the cost and amount of hardware needed to deliver tactile sensations to a user.

### Vibro-tactile stimulation

Percept sensation in the residual limb also can be generated using vibro-tactile stimulation elicited by mechanical vibrations of the skin (Tanaka et al., 2015). Different stimulation parameters (i.e., frequency and amplitude of the vibration) result in different types of sensory information such as proprioception (Kaczmarek et al., 1991; Mildren and Bent, 2016). However, other parameters such as duty cycle, shape, and pulse duration also can be used to convey different types of feedback (Cipriani et al., 2012; Dosen et al., 2016). The discriminating values of amplitude thresholds differ by prostheses and interface locations. Several sensory substitutions have been achieved through vibro-tactile stimulation, which includes the mapping of sensation from the prosthetic hand to the phantom hand for transradial amputees (Antfolk et al., 2013b; Papetti et al., 2016), the increase and decrease of texture roughness feeling on fingers (Asano et al., 2015), the sensation through stretching of the skin (Wheeler et al., 2010; Motamedi et al., 2017), the improvement in grasping force (Chatterjee et al., 2008; Cipriani et al., 2008, 2014; Saunders and Vijayakumar, 2011; Montagnani et al., 2015; Witteveen et al., 2015), the evoked sense of proprioception by the vibrations of a tendon (Thyrion and Roll, 2010), an object manipulation (Rombokas et al., 2013), the sense of embodiment (D'Alonzo et al., 2015; Ko et al., 2015), and the sensation on the surface of the skin (Kangas et al., 2017). The early devices were heavy and energy consuming. However, with the help of sophisticated electronics, tactile vibrators are now low powered and easily used for prosthetic applications. Generally, the use of vibrotactile feedback improves the user performance through a better control of grip forces and by lowering the number of errors in task execution. Unfortunately, due to the lack of intuitiveness and usability, the users don't feel comfortable with these types of indirect feedback systems.

### Modality-matched feedback

The most recent non-invasive technique used for conveying sensory information is the modality-matched feedback (see the mechano-tactile stimulation below): The input sensory stimulus must be the same modality as that of the sensory output. For example, for the sensation produced by touching, the prosthesis is required to be perceived as touch. This methodology is closer to naturally generated percepts. In theory, the coupling of non-invasive electro-mechanical devices with thermoelectric devices (e.g., Peltier cells) has made it possible to regain modality-matched touch sensations including contact, vibration, texture, temperature as well as normal and shear force/pressure. These coupled devices can be applied on the skin (Davalli et al., 2000), to the residual limb (Antfolk et al., 2012; Bjorkman et al., 2016), and to the chest and other body parts (Panarese et al., 2009) for achieving modality-matched touch sensations. This technique as applied for restoration of proprioception is inherently a challenge for engineers and researchers, as the angle of the wrist or hand joints is required to be manipulated to another intact joint in order to match the modality.

Mechano-tactile stimulation is utilized as one of the modality-matched techniques, which uses the application of force on a residual limb to evoke sensory feedback. An increase of the force in the prosthesis brings a proportional increase of the force applied to the skin in the residual limb. This technique may be superior to vibro-tactile substitution in that it reduces the grasping force error of prostheses, thereby providing more accurate spatial sensory information (Patterson and Katz, 1992; Antfolk et al., 2013a). However, Antfolk et al. (2013b) explained that the inherent design constraints on the size, weight, and response time of an actuator reduce the impact of this approach. D'Alonzo et al. (2014a,b) used hybrid vibro-electro-tactile stimulation and found it to be an efficient approach for obtaining multi-channel sensory feedback.

Regarding all the modalities noted above, achieving a selective and stable interface remains a challenge. Hence, researchers have looked for other near-to-natural-sensation modalities, among which peripheral implants are popular these days. In the next section, we will review the progress made in the neuroprosthetics using implantable interfaces at neuraxis and we will explore peripheral nerve-machine interfaces used for recording and stimulation.

## Invasive methods of sensory feedback

In the indirect methods noted above, complete selectivity and longevity have not yet been achieved. These modalities are still unable to provide selective sensory information in a stable manner. To overcome this issue, the peripheral interface technology has advanced more in recent years, and researchers are now focusing more on invasive technologies (Navarro et al., 1998, 2005). Tyler and Durand (1997) focused on the fact that for those amputees who only lost sensory end organs (limbs), the peripheral nerves connecting the brain to the lost organs retain the normal functionality. Thus, through a synthetic activation of these pathways, perception of sensation can be achieved (Micera and Navarro, 2009). There are several locations at which the interaction with the residual somatosensory system can be established (Weber et al., 2012). Direct stimulation on the nerve has earned extensive popularity in providing sensory feedback to prostheses. Preclinical works on the approaches related to the brain (Bensmaia and Miller, 2014), dorsal root ganglion (Weber et al., 2006, 2011; Bruns et al., 2013), and intra-spinal microstimulation (Gaunt et al., 2006; Capogrosso et al., 2016) are progressing.

### Neural interface and advantages

In the twenty first century, two important developments have transformed the field of neuroprosthetics. The first is the improvement in bionic arms (or prosthetic limbs) that can replicate the functions of a natural human arm. The second is the enhancement of algorithms that decode the intended movements of an amputee from neuronal activities in the motor cortex area of the brain. By combining these innovations, it is now possible for a human patient to perform tasks with a bionic arm by thoughts alone (Collinger et al., 2013; Wodlinger et al., 2015). Most existing interfaces are either from the nerve or the cortex (Tabot et al., 2015). The restoration of somatosensation, either from cortical stimulation or peripheral-nerve stimulation, is required for effective bidirectional communication and a sense of feeling or embodiment (Dornfeld et al., 2016) for the patient. The essential need for touch in everyday life has led several research groups to develop different techniques for its artificial restoration. The necessary methodology entails stimulation of the peripheral nerve or a somatosensory area of the brain (S1) with trains of electrical pulses to evoke percepts that transmit information from the grasping object (Downey et al., 2016). Amputees or patients that use a bionic limb controlled through such a bidirectional peripheral-nerve-interface perceive the prosthesis as an integral part of themselves rather than as a piece of hardware attached to their arm (Marasco et al., 2011; Limerick et al., 2014; D'Alonzo et al., 2015). The dexterity of a bionic arm can be improved through the restoration of somatosensation by stimulating the peripheral nerve, because in some cases such a manipulation of an object, visual feedback is considered to be a poor substitute (Bensmaia, 2015). To obtain such a feedback, several peripheral-nerve-interface approaches have been devised over the past two decades.

As noted above, electrical interfaces can be established anywhere in the cortex of the brain to the end organ. Sometimes interfaces are penetrative and sometimes stimulation can be given externally. In a broad spectrum, evaluation of these interfaces can be based on selectivity and longevity: Longevity is the measure of stable interaction of the electrodes with the same population of sensory afferents over time, and selectivity is the measure of its interaction with specific parts of sensory afferents. For the evaluation purpose, computational models of tactile afferents also have been deployed, which can simulate a population of afferents, in real time, in milliseconds and with precision (Kim et al., 2016). Furthermore, stability can be defined as the duration that the information (related with the activity measured by the electrode) should remain constant over the life of the interface. Greater stability of a stimulating electrode results in a more natural feeling of an artificial touch and is also important in achieving longevity for sensory feedback. Meanwhile, a higher selectivity can be achieved through intra-neural implants and, along with that, stability over a longer period also can be achieved. To increase selectivity, penetrating array type interfaces are used. However, extra-neural implants might stimulate a population of axons.

An interface with a cortical region of the brain faces similar longevity/stability and selectivity challenges, but in some different manner from the nerve case. The fundamental problem in cortical interfaces is longevity (Warren et al., 2016): The neurons (or neuronal tissues) at the implanted surface and the electrodes (implanted interface) degrade over time (McCreery et al., 2010; Prasad et al., 2012; Kane et al., 2013; Chen et al., 2014). These changes can affect the stimulation and recording abilities of the electrodes for sensory feedback or decoding the attempted movements (Perge et al., 2013).

Peripheral nerve stimulation through different interfaces is the hot issue for restoring sensations. In the next section, we will explain the recent advances for peripheral-nerve interfaces. The functional properties, selectivity, and biocompatibility will be discussed along with the advantages and disadvantages of various peripheral-nerve interfaces. The techniques in wide use for restoration of sensory feedback through electrical stimulation will be highlighted, though some other potential stimulation techniques such as the optogenetic modality (targeted neural signaling) (Towne et al., 2013; Warden et al., 2014; Pisanello et al., 2016) and the infrared-light-based technique (Wells et al., 2007; Cayce et al., 2015) are excluded.

### Extra-neural interfaces

In general, two broad categories of peripheral-nerve-interfaces are extra-neural and intra-neural. The extra-neural (or extra-fascicular) interface with a circular shape that surrounds the peripheral-nerve is a noninvasive method to the nerve itself. The electrodes do not penetrate the protective sheath (perineurium), and thus are less invasive and minimize the disturbances to the neural tissue. The cuff interface is most common among the extra-fascicular types. This electrode configuration can provide several distinct stimulation/recording according to the contacts around the periphery of the nerve. The helical (Agnew et al., 1989) and spiral (Naples et al., 1988) type interfaces have been proven to be stable for decades in clinical applications (Fisher et al., 2009; Polasek et al., 2009). Their circular shape offers a disadvantage of minimal interaction with neural tissues. Tyler and Durand (2002) introduced the Flat Interface Nerve Electrode (FINE), which is another form of extra-neural interface that was developed to increase the surface area without penetrating inside the nerve and to maintain the naturalistic shape of the nerve. Its recording ability (Yoo and Durand, 2005) and selectivity of the periphery of the stimulated nerve have been demonstrated (Schiefer et al., 2013; Ortiz-Catalan et al., 2014). Since the electrodes and the nerve fibers are separated by the perineurium, higher stimulation currents are required to achieve sensations than the case of intra-fascicular interfaces (Grinberg et al., 2008). Leventhal and Durand (2003) have employed a higher value of current resulting in activation at subfascicle level and achieved a low selectivity, and also their results were not repeatable: Hence, a similar tactile perception, which is not a naturalistic pattern of neuronal activation, was evoked. During grasping with an intact hand, every afferent fiber perceived differently and responded as per the object' features that invaded the fiber's receptive field, while electrical stimulation through the electrode sometimes produced a highly unnatural feeling or paresthesia due to synchronous activation of a large population of axons. The temporal modulation of stimulation pulse trains somewhat mitigated the effect of tingling and paresthesia. A stable and selective configuration of FINE and spiral interfaces has been achieved in clinical trials for more than 3 years for individuals with limb loss (Tan et al., 2014, 2015). Selectivity also has been achieved in this way, but only on the periphery of the nerve, not up to the axonal level. The main drawback of extra-neural interfaces is their low selectivity. In fact, being wrapped around the nerve, the electrode records the whole electrical activity of the nerve. As noted above, in order to reach afferent axons from the periphery of a nerve, they must provide high stimulation currents as compared to the intra-neural ones. For these reasons, an intra-fascicular type that penetrates the nerve itself has been introduced.

### Intra-fascicular interfaces

As the name suggests, an intra-neural interface penetrates the protective sheaths. The least invasive designs such as the groove interface (Koole et al., 1997) and the slowly penetrating inter-fascicular nerve interface (Tyler and Durand, 1997) penetrate only the epineurium. These interfaces physically insert electrical contacts within the nerve. Afferent/efferent fibers are in reach of these types of electrodes, and recording/stimulation is not as difficult to achieve as in case of other modalities. These interfaces, unlike extra-neural ones, tend to have more contacts within the peripheral nerve. Among the most invasive penetrating intra-neural interfaces placed inside the nerve fascicles, there are the Longitudinal Intra-Fascicular Electrode (LIFE) (Yoshida and Horch, 1993; Dhillon et al., 2004; Thota et al., 2015), a conducting wire or polymer filament implanted longitudinally and laid parallel to the nerve fibers, and the Transverse Intra-fascicular Multi-channel Electrode (TIME) (Boretius et al., 2010) having aligned contacts perpendicular to the nerve, respectively. LIFE has some drawbacks that include (i) a fixed distance between the electrode and the nerve which limits its selectivity, (ii) the interface stiffness causes micro-movements that, in the long term, may damage the nerve. The thin film Longitudinal Intra-Fascicular Electrode (tfLIFE) is an upgraded form of LIFE. It has been fabricated on a micro-patterned polyimide substrate, allowing several contacts in one interface. Its flexible structure allows a better adaptation to the nerve shape preventing damage due to an excessive stiffness. Selective interfacing is possible with LIFE and tfLIFE, which acquire signals from a small number of axons (Kundu et al., 2014). They are less invasive than recent multichannel array type interfaces. Selective stimulation and recording have been achieved through TIME at both intra-fascicular and inter-fascicular levels (Badia et al., 2011a, 2016). Also, multi-electrode-array-based interfaces (Byun et al., 2017) have been developed recently to demonstrate its viability for achieving sensorimotor information from the peripheral nerves. These interfaces have great benefits for individuals who have suffered a limb loss. Moreover, another penetrating type of interface is the Utah Slanted Electrode Array (USEA). This type has varying electrode densities according to several designs proposed in the literature with multiple penetration depths (Branner et al., 2001; Ledbetter et al., 2013; Wark et al., 2013). They are inserted perpendicularly into the peripheral nerve leading to a higher risk of nerve damage. They require a lower current for activation of nerve fibers due to their close proximity to axons and fibers; significantly therefore, a small group of fibers can selectively be stimulated (Branner et al., 2004). In this chronic implantation, the stability of an electrode of 80% silicon has been demonstrated for 7-months (Lacour et al., 2016). Davis et al. (2016) have achieved an increased selectivity and a stable sensory feedback by chronically implanted USEA in the median and ulnar nerves of human, which leads to an intuitive and dexterous control of prosthetic fingers with sensory feedback in the future for bidirectional prosthetic control. Coordinated grasp and sensory responses by stimulating the peripheral nerves of a monkey was demonstrated with the implantation of USEA. Moreover, with short-term implantation of intra-fascicular electrodes, increasing stimulation thresholds have been observed (Boretius et al., 2010; Rossini et al., 2010). The reported number of sensory perceptions and the locations evoked by intra-neural interfaces are very similar to those of the long-term extra-neural approaches, which led Grill et al. (2009) to conclude that the two approaches are equivalent. One of their disadvantages is the tendency to damage the nerve by penetration, which reduces the long-term stability. Nerve stimulation and excitation generated in the motor fibers can cause contraction of residual muscles, which may result in an obstructed control of bionic arms/prostheses.

### Regenerative peripheral-nerve interface (RPNI)

RPNI consists of an electrode and a residual peripheral nerve, which is neurotized by transacting the nerve and inserting the electrode in between them; it is an internal interface for signal transmission with the external electronics of a prosthetic limb. Effectively, it is a sieve made of silicon, ceramic or polymer with a large number of fine holes that is placed inside the transected nerve. The increase in growth rate of regeneration of individual or a group of fibers through those holes can activate selective stimulation and make it possible to record action potentials (Micera and Navarro, 2009; Sando et al., 2016) from individual axons or a small group. In principle, a high number of selective contacts can be achieved by reducing the size of the sieve along with an increased number of fine holes (Lago et al., 2007a). The neurotized interface serves as a biological amplifier and provides a long-lasting site for implantation. Studies revealed that the RPNI undergoes robust regeneration, neurotization, and revascularization (Urbanchek et al., 2016), and that signals are reliably transduced across the interface with longevity and reproducibility. Polyimide-based sieve electrodes have been shown to be biocompatible (Stieglitz et al., 1997) and stable over several months for *in vivo* implantation and testing (Navarro et al., 1998). Further experimentation with the interface revealed the stability and formation of new neuromuscular junctions within the muscles for improved function in amputees. Kung et al. (2014) proposed a new interface strategy to harness motor commands from transected peripheral nerves for control of a prosthesis. Also they demonstrated the 7-month viability of the RPNI. On the other hand, stimulation of a small number of regenerated fibers was shown feasible using the regenerative electrodes. By matching the regenerated nerve fascicles with the original receptive fields, an adequate feedback might be delivered. By targeting the fiber growth for a specific feedback of perception from a distinct neural population, there might be a possibility of generating a different fiber population that might not be required for the receptive field (Lotfi et al., 2011). Sensory feedback has been achieved from tactile and force sensors embedded in the prosthesis by stimulation of appropriate afferent fibers in the transected nerves. Another methodology to attain feedback has been proposed by Nghiem et al. (2015), which can be used for transferring sensory signals from a prosthetic sensor to the residual nerve. In this method, an insulated electrode placed on the surface of a muscle was used to stimulate the muscle, which then depolarized the afferent nerve within the muscle to provide sensory feedback. This new design can eliminate the problem of direct nerve stimulation that is inherent in intra-neural interfaces and also limits potential peripheral nerve damage. This sensory RPNI has the ability to transduce distinguishable and graded sensory signals across the peripheral nerve when being stimulated electrically. Although RPNI is still in an early stage of development, this technique has the capability to access sensory pathways and provide stable sensory feedback. A disadvantage of this modality is that the functional use of electrodes might require several months due to nerve regeneration. Furthermore, the nerve might degenerate with time, which will lead to a substantial loss of stimulation ability of the implanted electrode (Lago et al., 2005). A number of useful results have been obtained using the RPNI technique in experimental models (Ceballos et al., 2002; Ursu et al., 2016). However, some challenges remain, which hinder its clinical usability. The most important breakthrough of regenerative electrodes will be their implantation in the peripheral nerves of an amputee for bidirectional interfaces.

## Targeted reinnervation (TR)

**Figure 3:**
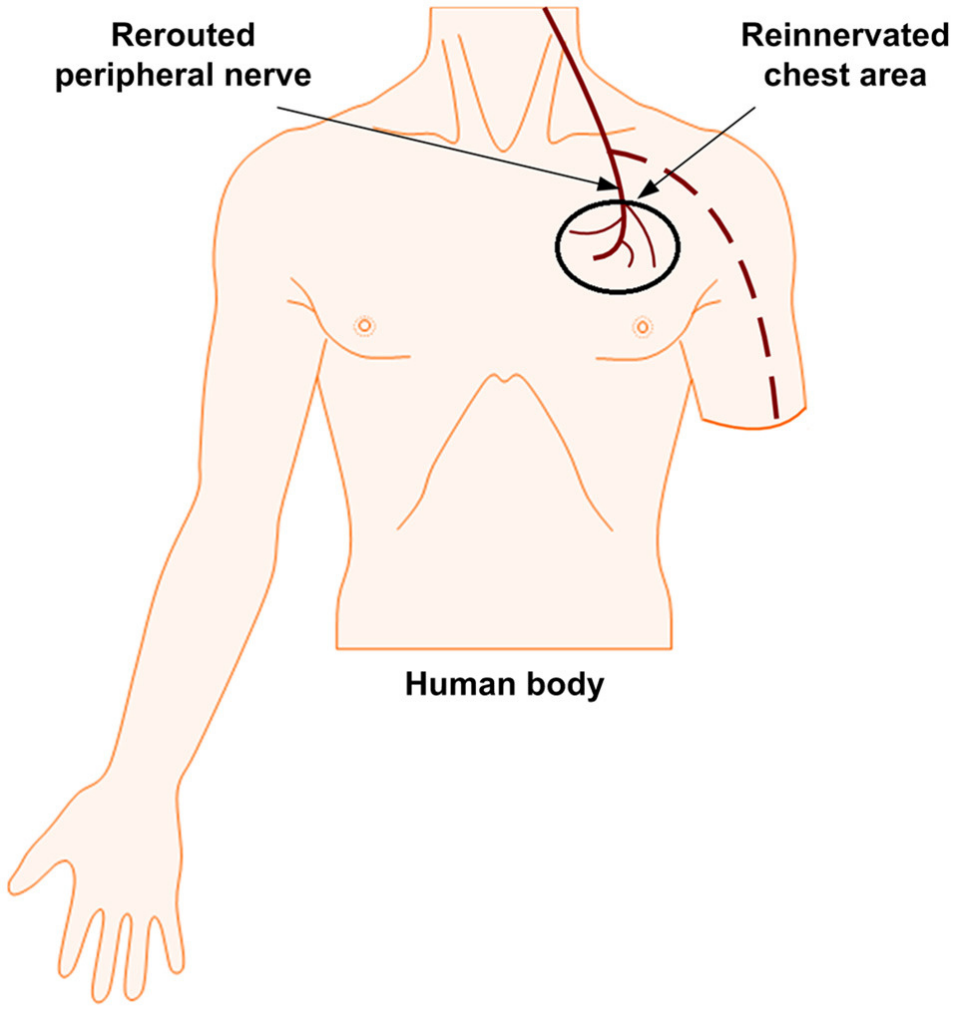
Targeted reinnervation: Stimulation site on the chest for sensory feedback.

TR is a nerve-machine interface that has been developed to make prosthetic control and feedback more intuitive. This method is considered to be fully neither invasive nor non-invasive; it lies in between due to the surgical requirement for its implementation. It has demonstrated a success in improving the motor control signals for both transhumeral and shoulder disarticulation amputation (Dumanian et al., 2009). It has also shown promising to sensory outcomes by using rerouted residual median, radial, and ulnar nerves. Instead of a direct electrical interface with the residual nerves in the arm, those in the arm can be moved to reinnervate the intact muscles in the chest, see Figure 3. This can help the transmission of sensory feedback and the attainment of electromyographic (EMG) signals by surface electrodes from the reinnervated site to control the prosthetic limb (Kung et al., 2013; Cheesborough et al., 2015). The EMG signals corresponding to the intended movement of a patient are generated from muscle contractions of redirected nerves. When the target skin of these patients was touched, they felt as if their missing limb was being touched (Kuiken et al., 2007a; Marasco and Kuiken, 2010). Studies of two amputees also have demonstrated a touch perception that aroused in the target skin: The amputees had a strong impression that the sensations arising from stimulation of the reinnervated skin site were projected to their missing limb. Furthermore, the sensory afferents remained stable for years after surgery. This methodology is being expanded in the field, evoking cutaneous or tactile sensations (Hebert et al., 2016) from the skin of reinnervated muscles. Using TR, sensation in the hand as evoked by a reinnervated chest skin along with other senses like touch, temperature, pain, and a limited graded force discrimination have been demonstrated (Kuiken et al., 2007b; Marasco et al., 2009, 2011; Schultz et al., 2009; Sensinger et al., 2009). There is a possibility of generating tactile feedback, but this technique is not naturalistic in restoring proprioception.

The advantages of TR technique, as stated by Hebert et al. (2014a,b), are as follows: (i) A long-term stable interface is possible, (ii) after rerouting of the nerves, there is no additional surgical procedure, (iii) the body is free of implanted interfaces, (iv) electrical stimulation evokes sensation to the reinnervated skin patch, and (v) there is no paresthesia or tingling. A series of results for patients who have undergone TR are; (i) non-invasive stimulation at the innervated site resulting in a generation of perceived sensory information (a cutaneous sensation) in the median nerve of the hand, (ii) effective detection of touch, pain, temperature, and proprioception to some extent, and (iii) stable reinnervated area for the detection of graded forces. TR does not only help in providing sensory feedback but also increases the size of the sites that can be used to control the prosthetic hand (Kuiken et al., 2004). However, Kristeva-Feige et al. (1996) have explained that there is a difficulty in restoring both sensory and motor control simultaneously because sensory-afferent signals are suppressed by motor-efferent commands. The proximity of the sites of sensory feedback and motor control can be considered as the major disadvantage of the TR technique. Also, the tactor array used for elicitation of sensory feedback at the TR location has only a limited ability in producing a wide range of sensation. The required calibration routine for taking the tactor array on and off has made this option rather difficult for daily use.

## Biocompatibility and neural recording/stimulation

Long-term stability and selectivity can be obtained if implanted interfaces are bio-integrated (or biocompatible) with the peripheral nerve. Foreign body reaction developed around the implants can affect the signal to noise ratio of the recorded motor commands and change the stimulation threshold values for sensory feedback. Therefore, existing interfaces and their fabrication have been considered as a practical choice for a prosthesis that intends to the recovery of sensorimotor abilities with characterization of long-term usability and biocompatibility. Such interfaces have offered a high-resolution means to access the information from the peripheral nerve by processing the multi-unit recordings and stimulations. Electrical stimulation of a single or a micro-electrode array has required current pulsations for eliciting action potentials. However, this approach is required to fulfill some conditions; (i) specifically designed interfaces for peripheral nerves, (ii) invasive enough to reach a target axon, (iii) ability to communicating bi-directionally, (iv) selective and stable electrical interfacing, (v) minimal tissue damages and influence of foreign body responses, and (vi) most importantly a biocompatible and biostable interface. Several types of interfaces proposed in the literature showed different material and chemical properties. Out of which, polymer based neural interface is popular. A conducting polymer is often used as a coating material on electrodes to increase charge-injection capacity for neural stimulation and to get high signal-to-noise ratio (SNR) for neural recording. Polypyrrole (PPy) and poly (3,4-ethylenedioxythiophene, PEDOT) are the most widely used polymer coatings for neural electrodes due to their biocompatibility. Different types of interfaces have been tested by several research groups on animals for checking their biocompatibility, recording and stimulating abilities for instance; Polyimide-based intra-neural implants (Wurth et al., 2017), USEA (Christensen et al., 2014), tfLIFE and tripolar cuff (Qiao et al., 2016), stretchable polymeric multi-electrode array (Guo et al., 2014), TIME-2 and TIME-3 with different chemical properties (Badia et al., 2011b), LIFE (Zheng et al., 2008), polyimide, platinum intra-fascicular electrodes (Lago et al., 2007b), and polymer-based longitudinal intra-fascicular electrodes polyLIFEs (Malmstrom et al., 1998; Lawrence et al., 2002).

As noted in the previous sections, several research groups have tested the capacities of intra- and extra-fascicular interfaces by implantation in the ulnar and median nerves to selectively evoke sensation in amputees through multiple electrodes. LIFE, TIME, FINE and the most recently used USEA have demonstrated interfacing capability with peripheral-nerves for sensory feedback. The details of the interface configurations and stimulation parameters in each human study are tabulated in Table 1. The artificial sensory signals are transmitted to the peripheral nerves by implanting electrodes providing a stimulated current that is proportional to the original input. By means of real-time sensory feedback, the amputee is able to move a prosthetic arm, apply a grip force via the recording of fine motor commands, and also feel a sensation without any audio and visual aids. Some studies have shown such ability of an amputee to differentiate objects according to their perceived characteristics (e.g., size, shape, and stiffness) and execute motor outputs such as grip strength (Horch et al., 2011; Raspopovic et al., 2014). The ability of object recognition and simultaneous encoding of sensory information for manipulation of grip forces are the excitatory developments for amputees. However, its widespread applicability is reduced by the limited number of studies and participants to date. Given the limited long-term data, it is significant to consider the physical conditions of the electrodes implanted in the peripheral nerves along with their capacity to provide a long-term stable interface (Yoshida et al., 2010). Structural changes of nerves can occur due to implantation. Fibrosis can hinder the response of an electrode and constantly reduce its performance. Implantation of tf-LIFE 4 in humans has shown a complete termination of sensory detection after 10 days of stimulation, which was due to the foreign-body response of tissue fibrosis (Rossini et al., 2010).

**Table 1:** Summary of human neural implant studies and modalities for restoration of sensory feedback.

**Study**	**Interface configuration**				**No. of subjects**	**Period of study**	**Sensory feedback modality**	**Selectivity and longevity**	**Stimulation parameters**					**Findings**
	**Type**	**Mode**	**Placement**	**No. of interfaces**					**Train duration**	**Pulse train**	**Pulse width**	**Current intensity**	**Frequency**	
Dhillon et al., 2004	Intra-neural LIFEs	Acute recording and stimulating	Severed median and ulnar nerves	2 each	8	2-days	Touch and proprioception	Selectivity only	500-ms	Monophasic, capacitively coupled, or biphasic, charge-balanced, rectangular	250-μs	Up-to 200-μA	Nil	Recording of volitional motor commands along with the elicitation of discrete, unitary and graded sensations of touch, joint movement and position
Dhillon et al., 2005	Intra-neural LIFEs	Recording and stimulation	Severed median and ulnar nerves	2 each	8	2-weeks	Touch, pressure and joint position		500-ms (5s between successive trains)	Charge balanced	300-μs	1–200 μA	Nil	Control of prosthetic arm has been improved with experience and training of several days. Showed stable sensation
Dhillon and Horch, 2005	Intra-neural LIFEs	Recording and stimulation	Median nerve	4–8 each	6	14-days	Grip force, touch and joint position			Charge balanced	300-μs	Nil	10–250 Hz (Position) 10–500 Hz (Pressure)	Direct neural feedback and control of an artificial arm having ability to judge finger force, static elbow, and finger position without visual feedback
Rossini et al., 2010	Intra-neural tf-LIFE4	Recording and stimulation	Median ulnar nerves	4 each	1	4-weeks	Touch		0.3-s	Rectangular cathodal	10–300 μs	10–100 μA	10–500 Hz	Real-time control of three motor movements and discrete tactile sensations achieved for first 10 days
Horch et al., 2011	Intra-neural LIFE	Stimulation	Median and ulnar nerves	2 each	2	9-days	Touch, finger position and force		290-μs	Biphasic	75-μs	Up-to 200 μA	30–200 Hz (proprioception) 20–170 Hz (touch)	Successful discrimination of objects using artificial tactile and proprioceptive feedback without visual or auditory cues
Raspopovic et al., 2014	Intra-neural TIMEs	Stimulation	Median and ulnar nerves	4 each	1	4-weeks	Touch sensation		Nil	Rectangular cathodal	Nil	160–240 mA	Upto 50 Hz	Bidirectional, near-to-natural control of a hand with stable tactile feedback
Ortiz-Catalan et al., 2014	Cuff	Chronic recording and stimulation	Ulnar nerve	1	1	More than 12-months	Tactile perception	Both	Nil	Single active charge-balanced biphasic	upto 500-ms	30–50 μA	8–20 Hz	Reliable, permanent bidirectional human-machine communication. Repeatedly similar in quality, magnitude, and localized sensory perceptions can be reproduced
Tan et al., 2015	Extra-neural FINE and Cuff	Chronic stimulation	Medial ulnar and radial nerves	2-FINE each (subject 1–2) 1-Cuff (Subject 2)	2	More than 24-months	Up-to 15-unique sensory percepts with some percentage of proprioception	Both	<30 s	Monopolar, bi-phasic, charge-balanced, cathodic-first square	24–60 μs (step size:10)	1.1–2 mA (step size:0.1)	10–125 Hz	Consistent threshold, impedance, and distinct percept areas have shown stability along with selective evocation of sensory perception without tingling or paresthesia
Davis et al., 2016	Intra-neural USEA	Chronic recording and stimulation	Median and ulnar nerves	1 each	2	4-weeks	Up-to 86-unique sensory percepts	Selectivity only	0.2–60 s	Current-controlled, biphasic, cathodic-first	200-μs	1–100 μA (step size:1)	1–320 Hz	Intuitive control of individual fingers of virtual robotic hand and decoded 13-movements offline and 2-movements online. Also evoked tactile perception with consistent signal-to-noise ratios and percept threshold
Oddo et al., 2016	Intra-neural TIME	Acute stimulation	Median nerve	1	1	4-weeks	Touch		Nil	Cathodic biphasic balanced square current	100-μs	100–160 μA	Nil	Reliable texture (coarseness of surfaces) discrimination with the help of touch perception

Additionally, some cortical implants like the Utah Electrode Array and the customized multi-channel stimulator for a cortical visual neuroprosthesis can be used for blinds (Ferrandez et al., 2007). Such stimulators have been proven to inject current (charge) in a safe and precise way in an acute animal experimentation. Another implant is the planar multi-electrode array for recording from a rat auditory cortex and visualizing a spatiotemporal structure of the cortical activities (Tsytsarev et al., 2006). The key to a further development of neuroprosthetics is to record simultaneous single-unit, multi-unit, and local field potential activity from multiple brain sites. Most recent study by Pothof et al. (2016) described the fabrication process of a chronic neural probe that has similar material and geometrical properties with that of clinical probes that enable recording of a single neuron activity at a high spatial resolution. These probes have successfully recorded single unit activities up to 26 days from the brain of a monkey that suggests its potential usefulness on human applications. Guitchounts et al. (2013) demonstrated the viability of long-term recording using a carbon-fiber electrode array, which have provided stable multi-unit recordings over a time-scale of months.

It is evident from above discussions that more research into long-term interfacing implants for bidirectional control of prostheses is required. In the following section, some parameters that can help in selective neural stimulation for elicitation of distinct and graded sensations (sensory feedback) are discussed.

## Characteristics of stimulation

Spikes are responsible for conveying information in the axons of a nerve, and the spike rate or frequency is involved in transmitting sensorimotor commands in a single axon (Tyler, 2015). Electrical stimulation in the form of spikes has been given to the residual stump nerve of a patient through typically implanted electrodes, and psychophysical judgments and verbal reports were gathered (Polasek et al., 2009; Schiefer et al., 2016). Stimulation through different electrodes is one way of manipulating evoked sensory percepts. Different populations of afferents can be activated through these individual electrodes, which can have distinct receptive field locations (Saal and Bensmaia, 2014) and different sub-modality compositions of sensory afferents (mix of slowly adapting (SA), rapidly adapting (RA), and pacinian corpuscles (PC) afferents). Thus, by evoking sensations through different electrodes at different locations in a phantom or residual limb, the field of projections of active fibers, which is the area where sensation is felt, can be determined. These sensations can also be determined through a sub-modality composition of activated fibers. For instance, if afferent populations of SA, RA, or PC fibers are stimulated, the evoked sensation will be of pressure, flutter, or vibration, respectively (Torebjork et al., 1987).

Moreover, the pulse shape is of extreme importance. The electrical pulse is transmitted to the nerve fiber using implanted electrodes. The field of pulse magnitude decays as the distance from the implanted electrode increases. Rattay and Aberham (1993) concluded that nerve activation is proportional to the rate of change of the voltage along the axons. Therefore, large, myelinated and closer axons can be activated by square pulses before small, unmyelinated and distant axons. A ramp pulse can be used to activate distal axons before closer ones (Grill and Mortimer, 1997; Grill et al., 2009), while a quasi-trapezoidal pulse have been employed for recruitment of smaller axons before larger ones (Fang and Mortimer, 1991). In implanted devices, exponential-shaped waveforms, utilizing minimal energy, can be used to minimize the power requirement for activation of larger and closer axons (Wongsarnpigoon and Grill, 2010; Krouchev et al., 2014).

As explained above, providing stimulation using different electrodes can elicit sensation of several different qualities. In addition to the closeness of intra-neural interfaces physically, another powerful tool is the design and manipulation of the spatiotemporal electro-magnetic field using extra-neural interfaces. These interfaces, with the help of multiple electrodes (Schiefer et al., 2008), generate an electrical field, the variation of which can change the activation areas of sensory percepts on an artificial hand/arm (using controlled inputs). The standard nerve stimulation paradigm is a train of identical, charge-balanced, square electrical pulses characterized by pulse amplitude, pulse width, and pulse repetition period (or inter-pulse interval). Traditionally, these three parameters are time-invariant and fixed in value. The intensity of a pulse can be manipulated in three different ways by varying pulse width (Tan et al., 2014), pulse frequency (Dhillon et al., 2005), and pulse amplitude (Raspopovic et al., 2014). It ranges from the lowest charge that can evoke a sensation all the way up to the value that elicits unnatural sensation (or paresthesia). By alteration of these stimulation parameters, sensations of varying quality can be evoked. The details of stimulation parameters used in different human studies for elicitation of sensory feedback are tabulated in Table 1.

In somatosensory applications, intensity is proportional to the variation of the stimulation frequency, and the resulting sensory perceptions can be changed with bursting pulse trains (Tan et al., 2015). In the patterned frequency paradigm, synchronous activity in a population of axons can be generated by maintaining the strength of the stimulation pulse constant. However, this cannot help in distinguishing complex sensory information. For generation of a patterned fiber activity, several modalities such as patterned field distribution of stimulation and patterned stimulation intensity have been employed. In each paradigm, non-synchronous activity in a population of axons is generated by creating a shift in the field between stimulation pulses. This information, if controlled properly, can be utilized for the restoration of several different somatosensory percepts. The nerve stimulation has been shown to induce different types of sensations; for instance, proprioceptive and touch sensations. Complex qualities of touch such as vibration on the skin, tingling, tapping, stinging, brushing, and itch have been observed during experimentation (Tan et al., 2014). Stimulation can infrequently evoke proprioceptive sensations, for example, sensations of movement of a finger or joint or a specific hand configuration. Systematic study of percept qualities and gradedness of these sensations have not been completed yet. Data on contact forces between the perceptually available object and the skin are required for grasping and manipulating an object (Johansson and Flanagan, 2009), because too much force is likely to damage the prosthesis or the object, while too little force can cause slippage (Witney et al., 2004). In previous studies, contact force has been manipulated through the intensity of stimulation; hence the greater intensity and the greater contact force.

## Restoration of somatosensory feedback through cortical stimulation

Although this review has focused more on peripheral-nerve recording, stimulation, and the methods adopted for encoding, we will briefly address the stimulation issue in the cortical region for restoration of somatosensory feedback. The field of neuroprosthetics has reached an age of maturity. When similar questions need to be addressed, different strategies are pursued (Courtine and Bloch, 2015). Elicitation of percept by electrical stimulation was first demonstrated by Wilder Penfield in 1937. They showed that somatosensory cortex (S1) neurons are organized into separate columns representing different regions of the body (Penfield and Boldrey, 1937). They later induced tactile sensation by applying electrical stimulation to the somatosensory area of a neurosurgical patient (Rasmussen and Penfield, 1947). The locations of percept areas on a body are systematically the same as those required on the cortical surface of the brain for stimulation (Rasmussen et al., 2009). The neurons that can react to similar types of stimuli have their functional columns in area S1. Studies also have found evidence for S1 regions that encode sensory information for individual digits of a hand along with different types of receptors within those digits (Merzenich et al., 1983; Sur et al., 1984).

Intra-cortical microstimulation (ICMS) has been applied to primates having the pulse frequency corresponding to the evoked perception of cutaneous flutter (Romo et al., 1998), which depicts the temporal configuration of pulse-train shape for elicitation of different sensations in a systematic mode. Recently, several studies have demonstrated the characteristics of ICMS by varying the parameters of stimulation for sensory feedback and recording of voluntary movements in primates (Fitzsimmons et al., 2007; O'Doherty et al., 2011; Zaaimi et al., 2013; Higo et al., 2016) and rodents (Fridman et al., 2010). Another important breakthrough is the first bidirectional brain-machine interface in which a signal from the motor cortex of a non-human primate was able to control the cursor, while stimulation is applied simultaneously to the brain area S1 to give sensory feedback on the movements, though the primate had to go through a learning process for mapping the afferent interface (Rajan et al., 2015; Tabot et al., 2015). These research advances have highlighted several methods of evoking broader ranges of near-to-natural sensations of touch and proprioception (Blank et al., 2010), with the hope that they will prove applicable to individuals who have lost limbs and/or senses. Somatosensation including proprioception is an essential element of natural motor abilities. Without having the sense of proprioception, it is difficult to plan a dynamic limb movement (Sainburg et al., 1995); indeed, an experiment on a monkey with a created lesion in S1 showed uncoordinated finger movements (Liu and Rouiller, 1999).

Many research groups are trying to use biomimetic approaches to achieve the sense of touch and proprioception integral to everyday behavior (Saal and Bensmaia, 2015). However, at present, large-scale neuronal activation relating specifically to the elicitation of detailed desired responses is not possible, due to the sensitivity of the brain. In other studies, ICMS pulse trains have been transferred through implanted electrodes (UEAs) in a monkey for restoration of sensory feedback (Bensmaia and Miller, 2014; Chen et al., 2014; Kim et al., 2015). In one study on rodents, the implementation of a biomimetic approach was tested by applying ICMS to the barrel cortex using micro-wires for the generation of artificial tactile percepts to navigate around a virtual target on the screen (Venkatraman and Carmena, 2011; O'Connor et al., 2013). With the help of this artificial sensation, the rats were able to find the virtual objects in order to obtain a reward with improved accuracy after training. Another experiment was conducted, in which the task was to detect the pulses of ICMS over a variable time interval. The rats were able to differentiate between pulses of ICMS and distractor mechanical stimuli to replicate the target stimulus.

ICMS can also be employed to deliver localized tactile percepts with natural features as well as in human-brain-computer interface applications for better control using area S1 (Collinger et al., 2013; Wodlinger et al., 2015; Downey et al., 2016). An experiment on the biomimetic method in non-human primates was conducted by delivering ICMS to area S1 in order to convey sensory information on the location of a contact in the hand; it was found that the receptive field of ICMS was highly localized to elicit percepts on the hand or even fingertips (Tabot et al., 2013). Various combinations of receptive fields can be used to signal which area of a prosthetic limb is in-contact with an object. The response of a sensor placed on the prosthesis can be used to activate neurons in area S1 with corresponding receptive fields. These approaches and advances can be served as a basis for improved somatosensory feedback in amputees (or tetraplegic patients). In patients, either a limb is missing or its connection with the brain has been lost, though the brain area responsible for tactile stimulation or sensory feedback for the corresponding limb is still intact (Flesher et al., 2016). These patients often feel a sensation at the residual limb (Ramachandran and Hirstein, 1998). One approach is to ask the patient how and where he/she feels sensation while delivering ICMS; then, the force with which an object is grasped with a prosthesis can be controlled by sending feedback through pulse trains of ICMS that elicits percepts of the correct magnitude (Berg et al., 2013). Task-specific somatosensory feedback has been generated in humans using cortical stimulation via electrocorticographic electrodes (Cronin et al., 2016). In the future, patients will be able to learn the meaning behind each combination of stimulation patterns with several types of sensory modalities and, by using that information, operate the prosthesis in more natural and effective ways. Above all, this method of evoking sensation is sensitive for an amputee, as all surgery and stimulation requirements are directly related to the brain, which is the most sensitive human organ, especially for those who have suffered from sense and/or limb loss. Therefore, peripheral interfaces have an edge over cortical interfaces.

## Current prosthetic technology

A substantial progress has been made over the past few decades in the neuroprosthetics technology. There are two types of prosthetics: Myoelectric- and body-powered prosthetics. Body-powered prosthetics have the advantage over myoelectric-powered ones, because an amputee can touch or feel the interaction with objects through the body harness operating the prosthesis (Carey et al., 2015). Battery-powered myoelectic prostheses, on the other hand, are based on muscle signals generated from the residual limb of an amputee through surface electrodes. These signals are transmitted to the actuators of the prosthetic limb to perform programmed movements. To evaluate prosthesis user functionality, several protocols have been developed (Chadwell et al., 2016). Pattern recognition control of myoelectric prostheses is also getting popularity due to its better classification of EMG signals (user intentions) using various methods, i.e., linear discriminant analysis classifier, tacit learning system, deep learning with convolutional neural network (Adewuyi et al., 2016; Atzori et al., 2016; Fani et al., 2016; Oyama et al., 2016), etc. Among the most recent achievements is the battery-powered myoelectric prosthesis with 16 degrees of freedom comparable to natural hand motions (Cipriani et al., 2011). Nonetheless, they provide less sensory feedback than body-powered prostheses. Myoelectric devices have relied only on auditory and visual sensory feedback, while body-powered devices rely on contrast and pressure, and the grip-strength information is transmitted through the hand using a cable and interfacing system. In some cases, the sensory feedback might not be the same as provided by the user's visual experience, but does provide a sense of control and connection to the device. Fifty percent of users do still prefer body-powered prostheses, possibly for improved feedback or referred sensation (Biddiss et al., 2007). The developments in volitional motor control have outpaced the advancements in sensory feedback for better control of prosthetic arms (Parri et al., 2017). Optimization of sensory feedback can provide better results, which include the restoration of a sense of touch that allows amputees to experience the world in a natural way and that also helps in allowing the bionic arm/prosthesis to be incorporated into their body image (Tyler, 2015). Along with visual feedback for monitoring of motor commands, the prosthesis must have tactile and proprioception feedback. Feeling the prosthesis as a part of his/her body has strong psychological benefits for patients. In any case, choosing prosthetics is always a tradeoff between sensation and function.

In comparison to EMG based control, the BCI is also getting popularity for control of prostheses in a non-invasive method. Electroencephalography (EEG) and functional near-infrared spectroscopy (fNIRS) are two major modalities used for BCI (Hong and Khan, 2017). EEG-based BCI mostly uses steady state visual evoked potentials and P300 for control applications (Turnip et al., 2011; Turnip and Hong, 2012). In comparison to EEG, fNIRS has lower temporal resolution. Still fNIRS-based BCI are effectively used for control applications (Santosa et al., 2013, 2014; Hong and Santosa, 2016). To overcome the issue of lower temporal resolution in fNIRS (Bhutta et al., 2015), hybridization (Khan and Hong, 2017), initial dip based feature extraction (Hong and Naseer, 2016; Zafar and Hong, 2017), and bundled-optode schemes are introduced for rehabilitation (Nguyen and Hong, 2016; Nguyen et al., 2016). However, the BCI modalities only decode the brain signals using active, passive, and reactive tasks (Khan and Hong, 2015; Hong et al., 2017; Liu and Hong, 2017). The current BCI systems do not have the ability to decode movement intention, instead they use imagination based tasks for control (Hong and Nguyen, 2014; Khan et al., 2014; Hong et al., 2015). Thus, for an amputee, the invasive nerve implants may be a more effective solution to BCI in comparison with EMG-based control.

The technology has been transferred to applications involving direct interfacing with peripheral nerves for intuitive control and sensory feedback. Different peripheral nerves can be activated through selective electrical stimulation. Hence, tactile or position sensations can be selectively and focally elicited without associated pain, while motor signals to extrafusal fibers are recorded more easily than those to intrafusal. The advancement in new peripheral interfaces has capitalized on this property to evoke touch perception without eliciting pain. Repeated clinical demonstration with replicated results has been achieved for peripheral interfaces (Schiefer et al., 2016). Bensmaia (2015) and Tyler (2015) concluded that many challenges are ahead before selective activation of all receptors. For instance, thermoreceptors for temperature feedback without simultaneous activation of others are required to be achieved yet. Several options are still required to be explored for nerve-machine interfaces (Rutten, 2002).

## Discussion

Upper-extremity amputations are usually associated with significant disabilities. Daily living activities are either no longer possible or require additional effort and time. This high level of disability serves to emphasize the obligation of the engineering research community to investigate, develop, and achieve innovative technologies for upper-limb prosthetics that can improve the quality of life. In fact, several advances already have been made in the upper-limb prosthetic technologies: battery- and body-powered, mechanically complex, and multifunctional prosthetic arms are now commercially available: However, there remains a lack of truly effective control and sensory-feedback interfaces.

As alluded to in the previous sections, natural sensory feedback in prostheses is highly desirable for amputees. A stable sensory-feedback system can enhance the control of prostheses. In fact, for a clinical use, a stable interface that is capable of selectively evoking several sensory percepts at several locations is indispensable. However, challenges to produce selective stimulation for chronically stable and long-lasting clinical applications for using peripheral nerve interfaces are still remaining. Although selectivity can be achieved through interfaces that penetrate the nerve for stimulation of one-to-one axons, this is with the cost of increased invasiveness. Additionally, whereas the modern prosthetic technology has achieved greater selectivity through regenerative, intra-fascicular and microelectrode-array-type interfaces, the problems are to test its biocompatibility and long-term stable recruitment in humans. These technologies have advanced the field of neuromodulation, but most human studies shown in Table 1 are of one or less than 1 month duration, which are helpful only in providing initial findings/data. Desirable features of peripheral nerve interfaces are low stimulation currents, selectivity, longevity, and long-term stability for stimulation/recording. These properties depend on the proximity of the exposed electrode surface to the target nerve fibers. Stability and selectivity have been achieved for longer periods of time, for instance, more than 3 years using the cuff-type interface (Tan et al., 2015). Therefore, more experimentation and changes in the design of implanted interfaces can provide selectively long-term graded sensation, tactile perception, and a sense of embodiment to improve the quality of prostheses. The non-invasive techniques in prostheses have been widely used in myoelectric (battery-) and body-powered prostheses. Their major advantage is the indirectness in communicating with the amputee (or patient), which means that they are non-invasive. However, these types of prostheses also have a severe disadvantage in that they lack wide sensory feedback. Some other modalities, such as the electro-tactile, vibro-tactile, and modality-matched feedback types discussed earlier do not provide appropriate feedback to an amputee and are less intuitive. In these cases, usability of these prostheses is decreased and the cognitive workload of the patient or amputee often increases.

With these considerations in mind, several groups have started developing invasive-type interfaces for prostheses that acquire signals and also provide sensory feedback directly from/to the peripheral nerves, which provides the amputee with a sense of perception and embodiment. An ideal bidirectional nerve-machine interface for prosthetic applications was shown in Figure 1. For its achievement, several methodologies have long been studied, based on which the most important goal is to design an interface that is stable and biocompatible and that can selectively acquire signals from the peripheral-nerve. The most recent study of Wurth et al. (2017) has demonstrated the selectivity, stability, longevity, and biocompatibility of intra-neural type interfaces. Additionally, the same nerve-machine interface is responsible for providing sensory feedback through stimulation that depends upon the area of the prosthetic hand that is in contact with an object. As noted above, several types of interfaces necessary for the completion of this nerve-machine interface have been designed, tested, and reported in the literature.

**Figure 4:**
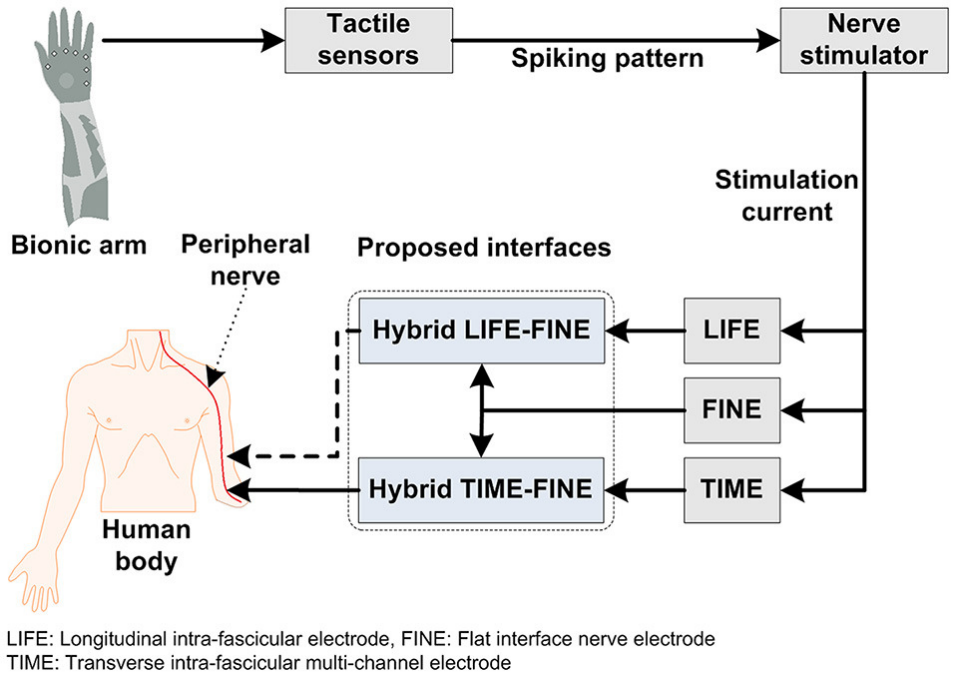
The proposed hybrid stimulation scheme for enhanced selectivity and longevity.

We propose a hybrid scheme in Figure 4, whereby two interfaces are added at a time. It is evident, from the fact indicated in Table 1, that LIFE has achieved better selectivity at the fascicular level in the peripheral-nerve (Dhillon et al., 2004, 2005) and that long-term stability and selectivity has demonstrated using FINE (Ortiz-Catalan et al., 2014; Tan et al., 2014). Like LIFE, TIME has also demonstrated greater selectivity in the peripheral nerve (Boretius et al., 2010; Badia et al., 2011b; Kundu et al., 2014; Oddo et al., 2016). Although USEA has showed better selectivity, it incurs a nerve-damage risk greater than that with LIFE or TIME, due to its approximately 96 microelectrodes that penetrate the nerve. It is pertinent to mention here that TIME and LIFE have multiple electrodes in one direction only, while USEA has multiple electrodes in the 2D plane. As noted in the previous sections, a lower current is required with TIME, LIFE, and USEA, while more current is required with FINE to elicit sensory feedback. By using a combination of these interfaces, which is to say hybrid FINE-LIFE or hybrid FINE-TIME, both selectivity and longevity, with increased numbers of stable sensory percepts and sensations, can be achieved. Because of the penetrating nature of LIFE and TIME, they can provide contacts at the axonal level, and FINE may not damage the nerve, as demonstrated clinically in previous studies. FINE's more-than-3-year stable implantation and sensory percepts in humans has showed its viability, although it obtained only 15–20 sensory percepts without tingling or paresthesia. For an increased number of sensory percepts at different locations in a hand, multiple electrodes were required as demonstrated by Davis et al. (2016). This type of interface configuration, with multiple-electrodes inside the nerve, incurs a greater risk of nerve damage as well as biocompatibility issues. In this strategy for achieving selectivity at the fascicular or axonal level, the implantation of TIME and LIFE, along with the combination of FINE, will prove beneficial in providing more sensory percepts selectively with an increased number of recording contacts for bidirectional control of prosthesis. Although clinical demonstration of this concept has not yet, to the authors' best knowledge, been achieved, it might prove useful in delivering better results for wide, distinct and stable sensory feedback in the neuroprosthetic technology.

## Conclusion

In this paper, we have reviewed the state of the art of peripheral-nerve-machine interfaces for restoration of sensory feedback. We have also discussed all of the invasive and non-invasive modalities of bionic/prosthetic arms for intuitive control and sensory feedback. The ultimate goal of all these interfaces is to provide a prosthesis with the ability to serve as an actual near-to-natural-limb replacement. Such prosthesis must be able to perform routine tasks and provide discrete sensation to the amputee. The lack of sensory feedback in myoelectric prostheses or those incorporating indirect methods of stimulation for sensation is a key limitation relative to the achievement of full control. Many researchers have tried several methods for providing sensory feedback. Among them, the peripheral-nerve-machine interface has become widely popular by providing selective and long-term stable feedback with more or less invasive type interfaces. Ultimately, the proposed hybrid LIFE-FINE or hybrid TIME-FINE interfaces based on the reviewed studies will provide an improved selectivity and longevity for bidirectional control of bionic arms.

## Author contributions

UG conducted the literature survey and wrote the first draft of the manuscript. SK participated in revising the manuscript. KH has conceived the idea, corrected the manuscript, and finalized the work. All the authors have approved the final manuscript.

### Conflict of interest statement

The authors declare that the research was conducted in the absence of any commercial or financial relationships that could be construed as a potential conflict of interest.
